# Sclerotic Rings in Mosasaurs (Squamata: Mosasauridae): Structures and Taxonomic Diversity

**DOI:** 10.1371/journal.pone.0117079

**Published:** 2015-02-18

**Authors:** Momo Yamashita, Takuya Konishi, Tamaki Sato

**Affiliations:** 1 Department of Astronomy and Earth Sciences, Tokyo Gakugei University, Koganei City, Tokyo, Japan; 2 Royal Tyrrell Museum of Palaeontology, Drumheller, Alberta, Canada; University of Oxford, UNITED KINGDOM

## Abstract

Mosasaurs (Squamata: Mosasauridae) were a highly diverse, globally distributed group of aquatic lizards in the Late Cretaceous (98–66 million years ago) that exhibited a high degree of adaptation to life in water. To date, despite their rich fossil record, the anatomy of complete mosasaur sclerotic rings, embedded in the sclera of the eyeball, has not been thoroughly investigated. We here describe and compare sclerotic rings of four mosasaur genera, *Tylosaurus*, *Platecarpus*, *Clidastes*, and *Mosasaurus*, for the first time. Two specimens of *Tylosaurus* and *Platecarpus* share an exact scleral ossicle arrangement, excepting the missing portion in the specimen of *Platecarpus*. Furthermore, the exact arrangement and the total count of 14 ossicles per ring are shared between *Tylosaurus* and numerous living terrestrial lizard taxa, pertaining to both Iguania and Scleroglossa. In contrast, two species of *Mosasaurus* share the identical count of 12 ossicles and the arrangement with each other, while no living lizard taxa share exactly the same arrangement. Such a mosaic distribution of these traits both among squamates globally and among obligatorily aquatic mosasaurs specifically suggests that neither the ossicle count nor their arrangement played major roles in the aquatic adaptation in mosasaur eyes. All the mosasaur sclerotic rings examined consistently exhibit aperture eccentricity and the scleral ossicles with gently convex outer side. Hitherto unknown to any squamate taxa, one specimen of *Platecarpus* unexpectedly shows a raised, concentric band of roughened surface on the inner surface of the sclerotic ring. It is possible that one or both of these latter features may have related to adaptation towards aquatic vision in mosasaurs, but further quantitative study of extant reptilian clades containing both terrestrial and aquatic taxa is critical and necessary in order to understand possible adaptive significances of such osteological features.

## Introduction

Mosasaurs (Squamata, Mosasauridae) were a group of secondarily aquatic lizards that radiated worldwide approximately from 98 Ma to 66 Ma of the Late Cretaceous [1]. Their modifications to limbs and axial skeleton [2–4], along with a shark-like, bilobed tailfin and a streamlined body in derived forms [5], [6] indicate a high degree of secondary aquatic adaptation and that mosasaurs became obligatory swimmers earlier in such a process. Numerous mosasaur fossils have been found globally, in particular from the Smoky Hill Chalk Member of the Niobrara Formation in the North American Western Interior Basin where the large, hydropedal russellosaurine *Platecarpus* and *Tylosaurus*, and the basal-most hydropedal mosasaurine *Clidastes* were particularly common and are well represented in museum collections worldwide [7–9]. Despite the large number of mosasaur specimens, however, preservation of complete sclerotic rings is extremely rare, a fact that has long hindered our understanding of this structure at the fundamental morphological level across a variety of mosasaur taxa (e.g., [10–13]). In fact, only the presence or overall morphology of a partial sclerotic ring and/or ossicles was briefly mentioned in osteological descriptions of mosasaurs (e.g., [7], [10], [14–18]), and only a single study exists to date that describes a complete mosasaur sclerotic ring, which was assigned to *Mosasaurus hoffmannii* [13].

Morphological study of sclerotic rings has a potential to be a powerful tool in the paleoecological interpretation of the fossil reptiles. The sclerotic ring in reptiles is composed of a ring of thin bony plates, i.e., scleral ossicles, embedded within the sclera, around the pupil, and posterior to the cornea [19]. Sclerotic rings are present in turtles, lizards, and birds, and absent in crocodilians and snakes [14]. In extant lizards and birds, the ring is typically composed of 10–15 scleral ossicles, with most lizards possessing 14 [14], [20–22]. Hypothesized main functions of the sclerotic rings range from maintaining the shape of an eye [23], [24] to providing the point of muscle insertions for visual accommodation [14], [20], [25]. Previous studies on modern birds and lizards revealed strong quantitative correlations between the ring and some soft tissue structures in shape and size [26], [27]. Furthermore, albeit qualitatively, many studies have also shown a strong correlation between visual function and lifestyle among modern vertebrates, including life underwater [21], [28–30]. Such correlations have been applied in the study of fossil vertebrates, leading to the inference of nocturnality in dinosaurs [31], [32] and estimation of the diving depth of the ichthyosaur *Ophthalmosaurus* [33], [34]. It is reasonable to expect that our descriptive study may form the future basis to infer the visual function and lifestyle in mosasaurs, the last major clade of the giant Mesozoic marine reptiles that dominated the marine realm until the end-Cretaceous extinction event.

In this study, we first provide detailed anatomical description of nearly complete to complete sclerotic rings in four disparate mosasaur genera that are known from the upper Coniacian–upper Campanian horizons of the Western Interior Basin: *Platecarpus*, *Tylosaurus*, *Clidastes*, and *Mosasaurus* (e.g., [9], [35–38]). In doing so, not only will we examine morphological variability among these taxa, but we also will compare the variation with that in extant lizards [14]. Based on the newly gained insights into such variability, we will then discuss the potential taxonomic and adaptational significance of the sclerotic ring types we recognize in mosasaurs, in the context of global squamate and mosasaur phylogenies.

Institutional abbreviations—CFDC, Canadian Fossil Discovery Centre, Morden, Manitoba, Canada; FFHM, Fick Fossil and History Museum, Oakley, Kansas, USA; KU, University of Kansas Museum of Natural History, Lawrence, Kansas, USA; LACM, Los Angeles County Museum, Los Angeles, California, USA; NHMM, Natuurhistorisch Museum Maastricht, Maastricht, The Netherlands; NSM, National Museum of Nature and Science (formerly National Science Museum), Tokyo, Japan; TMP, Royal Tyrrell Museum of Palaeontology, Drumheller, Alberta, Canada.

## Materials and Methods

### Materials

**Figure 1:**
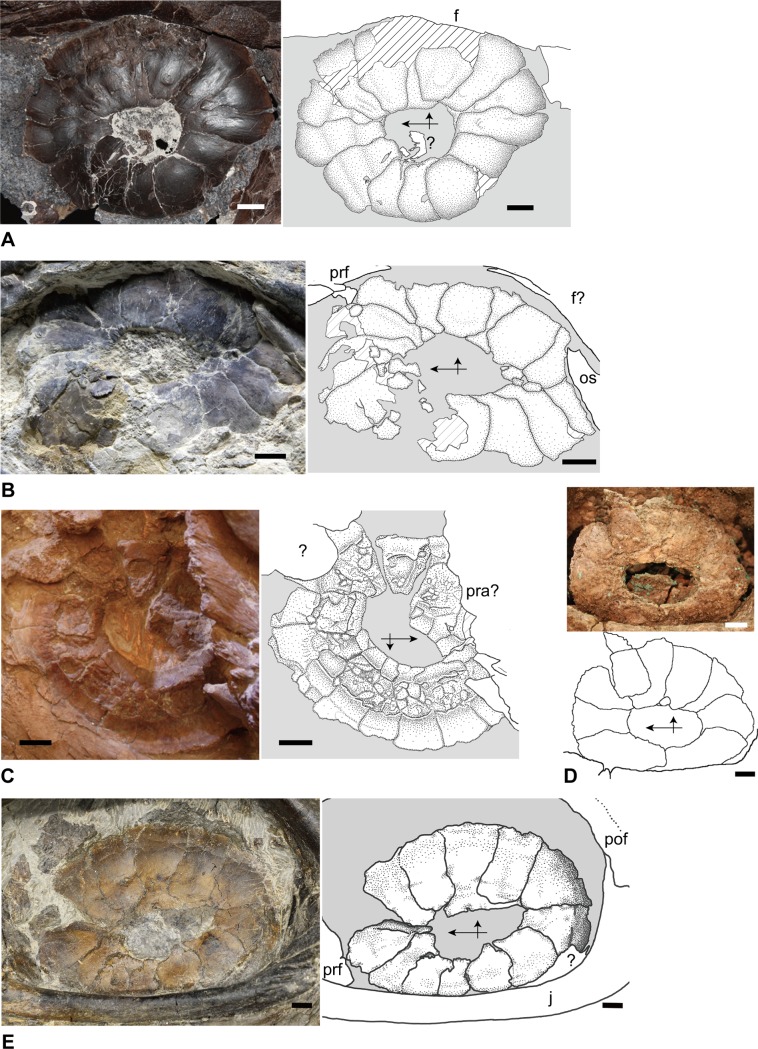
Sclerotic rings of five specimens of mosasaurs representing four genera. All showing left ring in outer view, where vertical arrows point dorsally and horizontal ones anteriorly. **A**, *Tylosaurus proriger* (FFHM 1997–10); **B**, *Platecarpus tympaniticus* (LACM 128319); **C**, *P*. *tympaniticus* (KU 1001); **D**, *Clidastes propython* (CFDC M74.10.06); **E**. *Mosasaurus* sp., cf. *M*. *missouriensis* (TMP 2012.010.0001). **Abbreviations**: **f**, frontal; **j**, jugal; **os**, orbitosphenoid; **pra**, prearticular; **pof**, postorbital frontal; **prf**, prefrontal. Scale bars equal 1 cm.

As all the specimens we examined in this study are curated at public institutions, no permits were required in conducting this study. As for mosasaurs, we examined five specimens representing four genera (Fig. 1) with the following provenances: CFDC M74.10.06 (*Clidastes propython*) is from the lower unit assigned to the *Baculites obtusus* ammonite zone of the Pembina Member exposed in southern Manitoba, Canada [36], [39]. The Pembina Member is the second lowest member of the Pierre Shale in the vicinity, and based on the ammonite zone, an earliest middle Campanian age is suggested for the lower subunit that yielded the largest number of marine vertebrate specimens, including CFDC M74.10.06 [36], [39], [40]. FFHM 1997–10 (*Tylosaurus proriger*) is from below Hattin’s Marker Unit 10 of the Smoky Hill Chalk Member in western Kansas is middle Santonian in age [41]. One of two specimens of *Platecarpus tympaniticus* (KU 1001) was collected in western Kansas in 1891 by the University of Kansas field crew under the supervision of Prof. Samuel W. Williston (see museum catalogues). This specimen is also from the middle Santonian chalk of the Smoky Hill Chalk Member [42], whereas another specimen (LACM 128319) is from an upper part of the Smoky Hill Chalk Member and is upper Santonian to lower Campanian in age [9], [43–45]. LACM 128319 was collected sometime in the 1960s in Logan County, Kansas by a local fossil collector M. C. Bonner, and was accessioned to the museum in 1969 [5]. TMP 2012.010.0001 (*Mosasaurus* sp., cf. *M*. *missouriensis*) was collected from the informal Muddy Unit 1 (lower part) of the Bearpaw Formation exposed south of Lethbridge, southern Alberta, Canada, and is late Campanian (ca. 74 Ma) in age [34], [46]. All the units were deposited in the Western Interior Seaway, which stretched across western North America from the Gulf of Mexico to the Arctic Ocean during the Late Cretaceous. The depth of the seaway in southern Manitoba is estimated to have been about 200–300 m [47], between 150–300 m in western Kansas [48], and between 50–70 m in southern Alberta (youngest in age) [49]. In addition, a complete sclerotic ring of Dumeril’s monitor *Varanus dumerilii* (NSM PO 391) is examined directly and described for comparison.

### Terminology

We follow [14]’s terminology for the three distinct ossicle types common in squamates, including mosasaurs: seen from outside, those that overlap both neighbors are the plus (+) type, those that are overlapped by both neighbors are the minus (-) type, and those with the one side overlapped by and the other side overlapping neighboring ossicles are the imbricating (i) type. In many lizards it is also common that adjacent ossicles show “S” shaped margins to overlap mutually, the state known as Verzahnung ([14]:25). We were unable to decisively identify Verzahnung in the mosasaur specimens we examined herein. We additionally follow the numbering system employed by [14] to describe the arrangement and the number of these differing types of scleral ossicles within each sclerotic ring, in which the plus type ossicle that lies temporal to (i.e., posterior to) the midventral ossicle is expressed as 1+, and the remaining plates are counted from 1+ temporodorsally (clockwise on the right side). For instance, the seventh ossicle counted from 1+ and that is a minus type is written as 7-, and this convention is followed for the remainder of this paper.

### Methods

**Figure 2:**
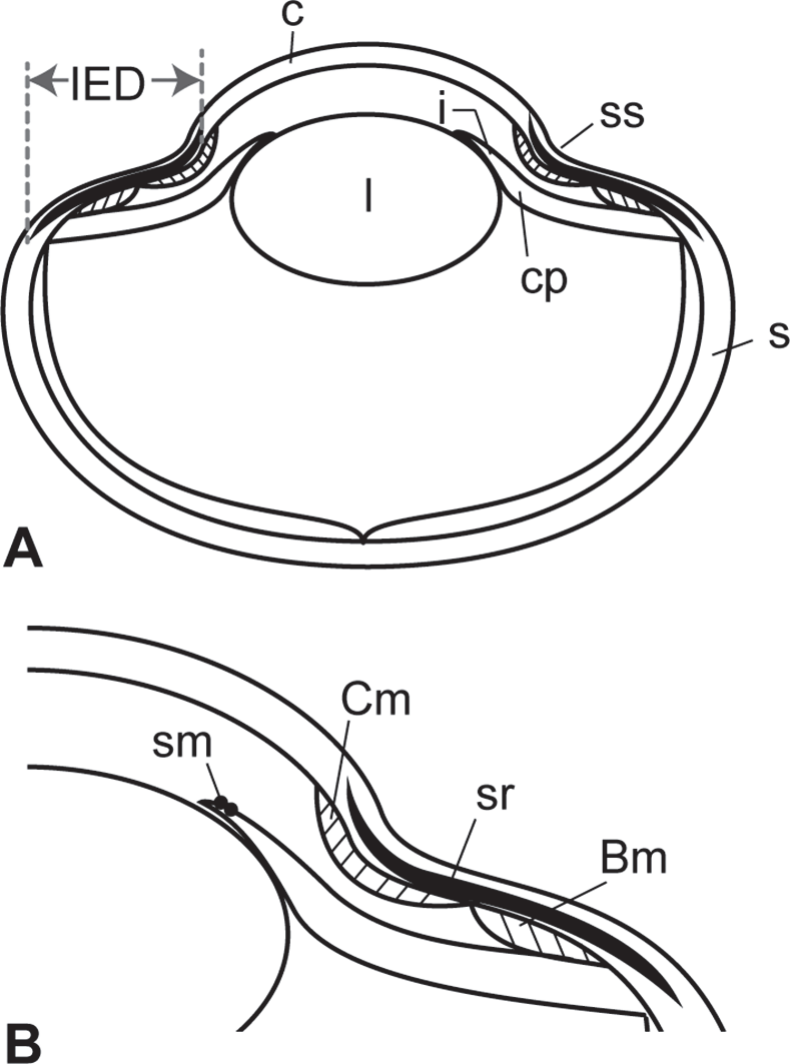
Structure of a generalized reptilian eye. **A**, Cross section of a lizard eye; **B**, Corneal section of an eye. **Abbreviations: Bm**, Brücke’s muscle; **c**, cornea; **Cm**, Crampton’s muscle; **cp**, corneal process; **i**, iris; **IED**, distance between internal and external borders of sclerotic ring; **l**, lens; **s**, sclera; **sr**, sclerotic ring; **sm**, sphincter muscle; **ss**, sulcus.

On FFHM 1997–10 and TMP 2012.010.0001, where possible, we measured the distance between the internal (corneal) and external (scleral) borders (IED), and the maximum width of each scleral ossicle (Fig. 2). Although the sclerotic rings of LACM128319, CFDC M74.10.06, and TMP 2012.010.0001 are ellipsoidal with their long axes trending horizontally, we ascribe this condition to postmortem distortion in the process of fossilization. We consider all mosasaurs possessed round sclerotic rings in life since sclerotic rings are in general round in living reptiles and birds, and because such a condition is equally common in various other mosasaur specimens (e.g., FFHM 1997–10, KU 1001, [13]). In Fig. 2, we show general structures of a reptilian eye to illustrate the anatomical features and dimensions mentioned in this study. All the measurements up to 150 mm were taken with digital calipers to 0.01 mm (to 0.02 mm with regular calipers in TMP 2012.010.0001), and with a tape measure when they exceeded 150 mm.

## Description and Comparison

**Figure 3:**
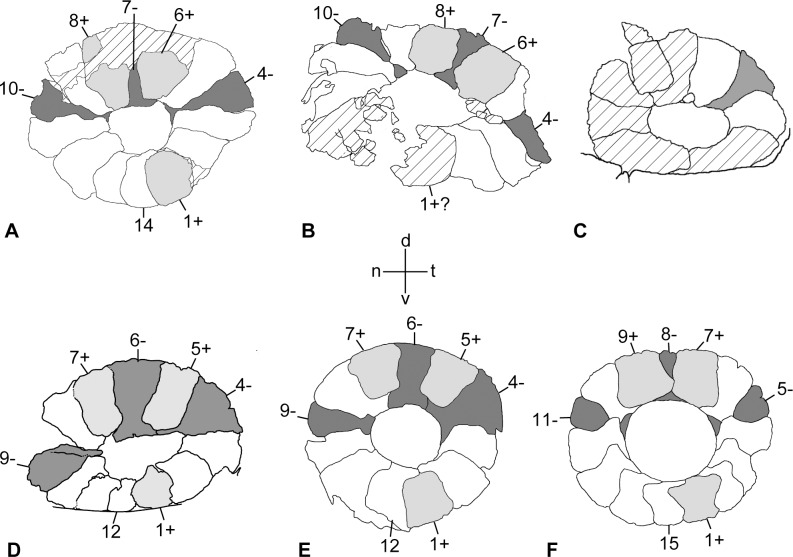
Observed arrangement of scleral ossicles in mosasaurs and a modern monitor lizard. **A**, *Tylosaurus proriger* (FFHM 1997–10, outer view of left ring); **B**, *Platecarpus tympaniticus* (LACM 128319, ditto); **C**, *Clidastes propython* (CFDC M74.10.06, ditto); **D**, *Mosasaurus* sp., cf. *M*. *missouriensis* (TMP 2012.010.0001, ditto); **E**, *Mosasaurus* sp., cf. *M*. *hoffmannii* (NHMM 2013001, ditto); **F**, *Varanus dumerilii* (NSM PO 391, ditto). Dark gray, light gray, and white indicate the ossicles of the minus-, the plus- and the imbricating-types, respectively. Except for final ossicle, only the plus- and the minus-type are numbered. Cross-hatching indicates broken parts. See main text for further explanation. Figures are not to scale. **Abbreviations: d**, dorsal; **n**, nasal; **t**, temporal; **v**, ventral.

**Figure 4:**
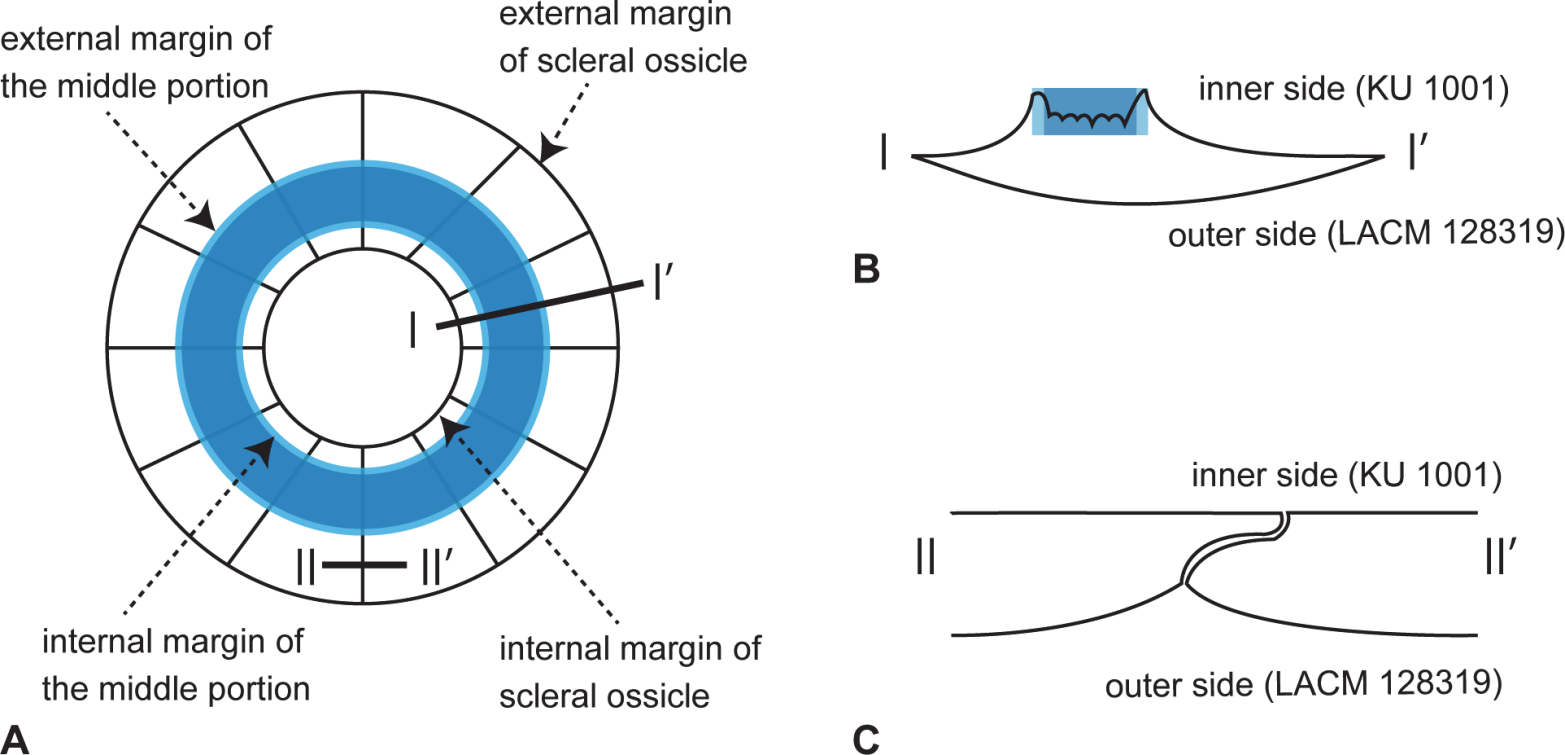
Difference of surface conditions between inner and outer side of the sclerotic ring in *Platecarpus tympaniticus*. **A**, interpretative drawing of inner side of the sclerotic ring based on KU 1001; **B**, cross sectional view of a single scleral ossicle along the line I-I’ in **A**; **C**, cross sectional view of a boundary of adjacent scleral ossicles along the line II-II’ in **A**. On the inner side, the raised middle area shaded in blue is roughened/asperate, resulting in obliterating ossicle boundaries.

As shown by [10] (Figs. 2–4), the outlines of the isolated scleral ossicles of the minus (-) and the imbricating (i) types in mosasaurs are generally similar, being rectangular with gently diverging lateral borders towards the external (scleral) end. On the other hand, a plus (+) type ossicle typically assumes a lozenge-shaped rectangle, being widest around the mid-height. There seem some exceptions to the foregoing generalization, which we will address below. In the following, we describe and compare five sclerotic rings pertaining to four genera and species of mosasaurs from the Western Interior Basin of North America. This is followed by description of the same for the extant monitor lizard, *Varanus dumerilii*.

### FFHM 1997–10—*Tylosaurus proriger*

The preserved left sclerotic ring is nearly round and the ossicles are arranged two-dimensionally, with individual ossicles snugly fitting with neighboring ones to complete the structure (Fig. 1A). This indicates that the ring experienced a minimum amount of distortion, and it is conceivable that the sclerotic ring in *Tylosaurus proriger* has been both circular and two-dimensional in life. The two-dimensional nature of the ring in FFHM 1997–10 is further accentuated by the fact that the internal (corneal) edge of each scleral ossicle lacks the outer-facing, sigmoid flexure that, upon forming the ring, supports a circular depression around the cornea, the sulcus (see the description of *Varanus dumerilii*) [14]. In common with many extant lizard taxa, a total of 14 scleral ossicles comprise the ring, each exhibiting gentle convexity on the outer surface. Of the 14 ossicles, three are the plus, three are the minus, and eight are the imbricating types, and within the ring they are arranged as: (1, 6, 8), (4, 7, 10), and (2, 3, 5, 9, 11, 12, 13, 14), respectively (Fig. 3A). Many of the extant lizard taxa sharing the total ossicle count of 14 with *T*. *proriger* also share this ossicle arrangement [14]. As the aforementioned arrangement suggests, the plus type ossicles are flanked by the minus type ossicle on one side and the imbricating type one on the other, or by a pair of imbricating type ossicles. In turn, the minus type ossicles are flanked only by a pair of plus type or that of imbricating type ossicles and not by the combination of both types. The imbricating type ossicles occur between all combinations of ossicle types except between two minus types and two plus types, as expected (Fig. 3A). The distance between the internal and the external borders (IED) differs slightly across the ring, even for the same type of ossicles. These distances are larger in the upper part of the ring of FFHM 1997–10, both absolutely and relatively, than those on the lower part (Fig. 3A, Table 1). In the dorsally situated ossicles, the IED is approximately twice as long as the ossicle width, while the same ratio is reduced to about 1.5 in the ventrally situated counterparts.

**Table 1:** The values of IED and width of each ossicle in FFHM 1997–10 (*Tylosaurus proriger*) and TMP 2012.010.0001 (*Mosasaurus* sp., cf. *M*. *missouriensis*).

FFHM 1997–10			TMP2012.010.0001		
Ossicle No.	IED	Width	Ossicle No.	IED	Width
1+	24.92	21.65	1+	24.00*	23.14
2i	23.18	22.55	2i	25.5	24.02
3i	34.93	-	3i	32.4	22.5
4-	37.02	17.31	4-	33.4	39
5i	38.19	18.26	5+	40	23.1
6+	-	-	6-	46.48	32.44
7-	-	-	7+	41.6	24.9
8+	40.2	-	8i	43.4	26.2
9i	38.15	-	9-	43.5	23.7
10-	-	-	10i	37.36	16.38
11i	36.02	-	11i	23.72**	17.34
12i	33.25	-	12i	17.00**	13.5
13i	26.1	-			
14i	-	-			

*…close estimation, **…incomplete (not fully exposed)

### LACM 128319—*Platecarpus tympaniticus*

As already figured in [5] (Fig. 2), the left sclerotic ring is well preserved in LACM 128319, in which possible melanosomes had been identified within the aperture [5]. The ring (Fig. 1B) is two-dimensional lacking any sigmoid flexures, and both the ring and the aperture are horizontally elongate due to deformation, more so than in KU 1001 (Fig. 1C). Fine grooves cover the entire external surface, while short, strong grooves are born along the internal margin in this specimen (Fig. 1B). The outer surface of the ring is smooth otherwise and slightly convex, both features shared with *Tylosaurus proriger*. At least 13 and possibly 14 ossicles comprise the sclerotic ring, consisting of eleven ossicles of known type—two plus, three minus, and six imbricating type—and two or possibly three additional ossicles, whose types we could not identify (Fig. 3B). Assuming that the midventral ossicle is plus type and the 13^th^ one imbricating type, the arrangement of three ossicle types becomes as follows: plus type = (1, 6, 8), minus type = (4, 7, 10), and imbricating type = (2, 3, 5, 9, 11, 12, 13) (Fig. 3B). Notwithstanding the uncertainty concerning the first and the 13^th^ ossicle type, the remainder of the ossicles indeed shares the identical arrangement to that of FFHM 1997–10 when 1+, 13i, and 14i were subtracted from the latter. Among the identifiable ossicles in LACM 128319, the following arrangements occur: the minus type ossicle between two plus type ossicles or between two imbricating type ossicles, the plus type ossicle between the minus and the imbricating type ossicles, and the imbricating type ossicle between the minus and the plus type or between the minus and the imbricating type ossicles (Fig. 3B). Other arrangements of ossicles confirmed in FFHM 1997–10 were possibly present but could not be confirmed. In contrast to FFHM 1997–10 however, the scleral ossicles that occur along the dorsal part of the ring appear to have shorter IEDs than those along the ventral section. At the same time, the external margins of the dorsal ossicles in LACM 128319 are still partly covered by the matrix, while further preparation cannot be performed due to the exhibited nature of the specimen.

### KU 1001—*Platecarpus tympaniticus* (Inner Surface)

Unique among the five examined specimens, this specimen shows the inner side of the sclerotic ring on the right side (Fig. 1C). The ring is two-dimensionally preserved, and it is slightly elongate along the horizontal axis. Some ossicles are fractured in the middle and this renders distinguishing true ossicle boundaries from broken lines difficult. Nevertheless, we confirmed the presence of at least 13 scleral ossicles, where at least one minus type ossicle, one plus type ossicle, and one imbricating type ossicle were identified.

Somewhat unexpected, the inner surface of the ring is not homogenously planar or smooth, where the middle portion of each scleral ossicle is raised and asperate (Figs. 1C, 4). The portions near internal and external margins of each ossicle are thin, gradually becoming thicker towards the raised middle portion forming a gently concave, smooth surface. This smooth surface is larger on the external side of the raised middle segment. The middle portion itself is depressed slightly, and its surface appears broken into irregular pieces, producing the asperate texture (Fig. 4). As on the outer side, short, radiating grooves occur along the internal margin of the ring on the inner side. In addition, the ossicles across the ossicle boundaries are structurally more continuous on the inner side, where, outside of the raised middle area, adjacent ossicles come in contact along planar surface. On the other hand, as each ossicle is gently convex on the outer side, weakly bulged ossicle surfaces meet with each other forming a shallow groove in between.

### CFDC M74.10.06—*Clidastes propython*

The left sclerotic ring is shown in lateral aspect (Fig. 1D). As in the other mosasaur specimens and in extant *Varanus*, overall the ring is two-dimensional. The internal edge of each sclerotic ring lacks a flexure as well, although selenite encrustation could have obliterated some details. As preserved, both the sclerotic ring and the aperture are longer than tall, likely having experienced some postmortem dorsoventral compression (see KU 1001 and LACM 128319 above). Selenite encrustation obscures ossicle boundaries particularly in the lower half of the ring; nevertheless, at least nine scleral ossicles could be counted (Figs. 1D, 3C). In CFDC M74.10.06, only two imbricating type and one minus type ossicles can be identified with confidence (Fig. 3C). Consistent with FFHM 1997–10, the distance between the inner and the outer borders of the scleral ossicles is greater in the dorsal part than in the ventral part of the sclerotic ring, even though the width of each ossicle could not be measured directly.

### TMP 2012.010.0001—*Mosasaurus* sp., cf. *M*. *missouriensis*

A total of 12 scleral ossicles comprise an overall two-dimensional left sclerotic ring of this late Campanian specimen, which most likely pertains to *Mosasaurus missouriensis* [38] (Fig. 1E). The outer surface of the sclerotic ring is smooth and gently convex from the external (scleral) to the internal (corneal) borders. Short, radiating grooves are born only along the internal margin of the two ventral ossicles (i.e., 11i and 12i), while the other plates are smooth. In a complete sclerotic ring from Maastrichtian strata of Belgium, consisting of 12 ossicles and assigned to *Mosasaurus hoffmannii*, such grooves are similarly restricted to the anteroventral quarter of the internal rim (10i–12i), while the rest of the ossicles are smooth on the outside [13]. These grooves are more widely distributed in LACM 128319, *Platecarpus tympaniticus*, occurring along the dorsal half of the internal rim of the ring. On TMP 2012.010.0001, many scleral ossicles exhibit a beveled internal edge, particularly on the ossicles 3i, 5+, 7+, 10i, and 11i, and none shows a sigmoidal flexure. As indicated in Fig. 3D, the plus type, minus type, and imbricating type ossicles are arranged as follows, respectively: (1, 5, 7), (4, 6, 9), and (2, 3, 8, 10, 11, 12). The same arrangement occurs in *Mosasaurus hoffmannii* [13] (Fig. 3E). In spite of the smaller total ossicle count of 12, TMP 2012.010.0001 exhibits all the ossicle alignment patterns possible for each ossicle type, including the two that are absent from *Tylosaurus proriger* and *Platecarpus tympaniticus*: i.e., one plus type ossicle (5+) is flanked by two minus type ossicles, and one minus type ossicle (4-) occurs between the plus type and the i type ossicles (Fig. 3D). As in FFHM 1997–10 (*T*. *proriger*) and CFDC M74.10.06 (*C*. *propython*), the IEDs for the dorsally situated ossicles are greater than those for the ventrally situated ones, where 6- is the largest and 12i is the smallest ossicles (Table 1). Comparing the plus type ossicles across the sclerotic ring aperture, for example, IED for 1+ is close to 24.00 mm and it virtually equals the plate width at 23.14 mm, while IED for 5+ is 40.00 mm and is nearly twice as long as it is wide (23.10 mm). Of note, 5+ and 7+ ossicles do not reach the sclerotic ring aperture, whereas the internal border of all the plus plates bears an apex. In contrast, the same border is either shallowly concave or nearly straight in the minus and imbricating type ossicles (Fig. 3D). Finally, both 5+ and 7+ are rectangular in outline, lacking the lateral expansion in the mid-height.

### NSM PO 391—*Varanus dumerilii*

**Figure 5:**
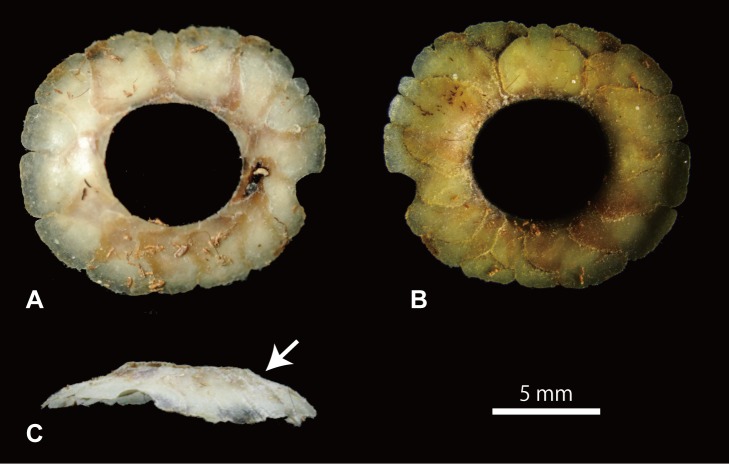
Sclerotic ring of a modern monitor lizard. **A**, outer surface; **B**, inner surface; **C**, lateral view. White arrow indicates the sigmoid flexure that supports a circular depression/sulcus which surrounds the cornea of the lizard eyeball.

The undistorted sclerotic ring is circular and nearly two-dimensional, consisting of 15 scleral ossicles that include three ossicles each of the plus and the minus type. The exact anteroposterior direction of the sclerotic ring cannot be determined as the ring was free from the orbit before this study, but based on [14] the ring is from the left side and shows the following ossicle arrangement: plus type = (1, 7, 9), minus type = (5, 8, 11), and imbricating type = (2, 3, 4, 6, 10, 12, 13, 14, 15) (Fig. 3F). In the upper portion of the ring, two ossicles of the plus type contact with each other across the minus type ossicle they flank (Figs. 3F, 5A). The feature that distinguishes the sclerotic ring of *Varanus dumerilii* most from those of any mosasaur specimens examined above is the distinct sigmoidal flexure near the internal edge of each scleral ossicle (Fig. 5C, arrow). When these ossicles form a ring, the resulting annular depression underlies the sulcus that forms along the sclera-cornea boundary [13] (Fig. 5 B, C). The inner surface is as smooth as the outer surface (Fig. 5B).

## Discussion

Arrangement patterns of scleral ossicles have thus far been investigated among a broad range of taxa of modern birds and lizards [14], [28]. However, there exist no previous studies on comparison of the arrangement pattern between extant and extinct taxa such as mosasaurs, the group that fully adapted to aquatic environment. By comparing sclerotic rings between mosasaurs and modern lizards, it must be possible to evaluate the variation in arrangement patterns of scleral ossicles in terms of aquatic adaptation, for example. Through such comparative studies, we may ultimately be able to identify sclerotic ring features associated with underwater vision. One of the functions of the osseous ring is to provide attachment points for muscles used for accommodation [14], and the way a vertebrate eye accommodates differ significantly between terrestrial and aquatic environments [19], [24].

### Arrangement Patterns of Scleral Ossicles in Mosasaurs

Sclerotic rings of the four mosasaur taxa allow us to compare both the specific and overall structures of the ring among these mosasaurs and with those of extant lizards for the first time. In particular, we newly identify the range of variation in the total ossicle count and arrangement among these mosasaurs, and add to the known morphological range for non-ophidian squamates [14], [50]. For phylogenetic frameworks, we refer to [51] for the relationships among major squamate clades inclusive of fossil taxa, and [8] for mosasaur phylogeny at lower taxonomic ranks. We note that [51] and [8] do not agree on the relationships within the Mosasauridae; we employed the latter because it is a comprehensive study including all mosasaur taxa described in this study and it also represents a view largely agreed upon by a majority of recent mosasaur studies.

For the total counts and arrangement patterns of scleral ossicles in mosasaurs, we used the russellosaurine *Tylosaurus* specimen (FFHM 1997–10), *Platecarpus* specimen (LACM 128319), and the mosasaurine *Mosasaurus* specimen (TMP 2012.010.0001) because their scleral ossicles are well delineated and their rings have been almost entirely exposed (Figs. 1, 3). Preservation of the only specimen of the basal mosasaurine *Clidastes* is less favorable and comparison is limited to the number of ossicles and overall morphology. As described above, both the total ossicle count and arrangement in the two russellosaurine genera proved almost identical, with slight uncertainty arising from the incomplete preservation of LACM 128319. If we assume the midventral ossicle in LACM 128319 to correspond with 1+ in FFHM 1997–10, then the alignment of consecutive 11 out of 14 (80%) ossicles of the latter is shared by the former as follows: 2i, 3i, 4-, 5i, 6+, 7-, 8+, 9i, 10-, 11i, 12i (Fig. 3A, B).

According to [14], [50], the following extant lizard taxa are known to possess 14 ossicles per ring with the identical ossicle arrangement to *Tylosaurus*: 5 genera of Gekkonidae, 17 genera of Iguanidae, 3 genera of Teiidae, 1 genus of Scincidae, 2 genera of Lacertidae, 3 genera of Cordylidae, and two genera of Xenosauridae. Furthermore, the arrangement of these 11 consecutive ossicles shared between *Tylosaurus* and *Platecarpus* is also shared by several species of *Varanus*, including *V*. *dumerilii*, with 15 ossicles per ring (Fig. 3A, B, F) [14]. These extant taxa which share the ossicle arrangement pattern with the two russellosaurine genera include basal and derived members of the Iguanidae [51], as well as a number of scleroglossan taxa. It is noteworthy that the Iguania and Scleroglossa are two major clades within the Squatama, and the Mosasauria (= Mosasauridae and closely related forms) according to [51] is the sister taxon to the Scleroglossa. Meanwhile, *Sphenodon*, the only living representative of the Rhynchocephalia that is the sister taxon of the Squamata, does not share this condition; it possesses as many as 16 ossicles including three plus type and minus type ossicles each [14]. The unique nocturnal adaptation of *Sphenodon* warns against the over-generalization in the foregoing sclerotic ring characters for the rhynchocephalian clade as a whole, but the well-established sister group relationship between Rhynchocephalia and Squamata [51], as well as the commonness of the arrangement of the 14 ossicles in many members of Iguania, Mosassauria, and Scleroglossa could well suggest that such a sclerotic ring condition be symplesiomorphic for Squamata. While a thorough testing of this phylogentic hypothesis requires an increased taxonomic sample size and is beyond the scope of our current study, we point out that those living squamate taxa sharing the said ossicle number and the arrangement exhibit diverse life habits, such as crevice-dwelling *Xenosaurus* and semiaquatic *Shinisaurus* [52]. The newly confirmed presence of this osteological character in the Late Cretaceous, obligatorily aquatic mosasaurs *Tylosaurus* (and most likely *Platecarpus*) is therefore not entirely unexpected.

Meanwhile, and somewhat unexpected, the aforementioned pattern is not shared with the mosasaurine mosasaurs examined in this study. Both the total ossicle count and the ossicle arrangement in *Mosasaurus* sp., cf. *M*. *missouriensis* (TMP 2012.010.0001) and *M*. *hoffmannii* [13] are identical (Fig. 3D, E) despite their temporal separation of about 10 million years, presumably showing some phylogenetic constraint. Both attributes are indeed distinct from those of Russellosaurina examined and any of the extant lizard taxa for which these characters are documented [14]. For example, almost all lizard genera with the ring consisting of 12 scleral ossicles as in *Mosasaurus* only possess two ossicles each of the plus and minus types (e.g., Agamidae) [14]. Iguanian *Phrynosoma* and *Holbrookia* are notable exceptions in possessing three ossicles each for the two types, but unlike *Mosasaurus* spp., their minus type ossicles are arranged as (4, 6, 8) instead of (4, 6, 9) (cf. Fig. 3D, E). One plausible evolutionary explanation for the *Mosasaurus* condition is subtraction of the 5i and one of the i type ossicles between 10- and 1+ in the configuration observed in *Tylosaurus* and in many other non-ophidian squamate taxa. The total number of dorsal ossicles and their morphology in the basal mosasaurine *Clidastes* (CFDC M 74.10.06) appear more comparable to *Mosasaurus* than the russellosaurine taxa examined (Fig. 3), although we need better specimens to confirm the details.

### Other Notable Sclerotic Ring Morphology

It is consistently shown among nearly/fully exposed sclerotic rings of FFHM 1997–10, CFDC M76.10.01, TMP 2012.010.0001, and *Mosasaurus hoffmannii* [13] that the sclerotic ring aperture in these mosasaurs is positioned slightly ventral to the center of the ring. In TMP 2012.010.0001, for example, the largest ossicle 6- on the dorsal side has the IED that is approximately twice as long as that of 1+ across the aperture on the ventral side of the ring. This is in stark contrast with the observation we made on *Varanus dumerilii*, in which the large aperture is found more or less in the center of the ring (Fig. 5A). It follows logically that the relative position of the aperture within the bony ring should be correlated with the position of the cornea in relation to the ring, and may have affected, to some degree, the visual field of those sea-going reptiles. Among squamates, the occurrence of ventrally positioned sclerotic ring apertures is not exclusive to mosasaurs (e.g., *Anolis* [14] (fig. 7i)). The inner side of a sclerotic ring of the *Platecarpus tympaniticus* specimen (KU 1001) is uniquely asperate in the middle, while the outer surface is overall smooth in the conspecific LACM 128319 (Fig. 4). The inner surface is clearly not asperate in modern *Varanus dumerilii* on the other hand (Fig. 5B), suggesting a possible functional significance of the difference.

Inside a vertebrate eye, scleral ossicles are embedded in a specific part of sclera that is attached to muscles for corneal accommodation of an eye, i.e. changing the focal length by deformation of the cornea (Fig. 2B) (e.g., [13, 24]). Accommodation of eyes is different between aquatic and terrestrial animals regardless of phylogeny, as the corneal refractive power is negated in water [53], [54]. In the eyes of secondarily aquatic vertebrates, the lens is more spherical and the cornea is flatter than the terrestrial eyes [19], [55], [56]. An aquatic eye accommodates by forward lens movement or by squeezing the anterior surface of the lens using robust iris sphincter muscles [19], [30], whereas a terrestrial eye accommodates by changing the shape of the cornea (i.e., corneal accommodation) and/or the lens (i.e., lenticular accommodation) [24], [57]. In the latter, the anterior and posterior segments of the ciliary muscle are used respectively, i.e., Crampton’s muscle and Brücke’s muscle, extending from beneath the scleral ossicles to the inner lamella of cornea and the ciliary body, respectively [14], [24], [29] (Fig. 2).

The difference of the condition in the inner surface between the observed *Platecarpus* and *Varanus* sclerotic rings may reflect the aforementioned difference in accommodation between aquatic and atmospheric media, where the raised and asperate inner surface of the former may have served as a major attachment site for muscles involved in changing the lens position in lieu of its shape in aquatic environments. At the same time, there exists a limited number of studies that established a relationship between the sclerotic ring and soft tissue structures of an eye, and hence activity patterns, in modern lizards [27]. Further investigation in living non-ophidian squamate taxa is necessary in order for us to infer paleoecology of fossil squamates, including mosasaurs.

## Conclusions

The sclerotic rings in four mosasaurid genera from the Late Cretaceous Western Interior Seaway of North America, *Tylosaurus*, *Platecarpus*, *Clidastes*, and *Mosasaurus*, were described and compared with one another and with those of extant lizard taxa, including *Varanus dumerilii*. The arrangement patterns of the scleral ossicles in *Tylosaurus* and *Platecarpus* are (nearly) identical to many modern lizard families, including and notably non-scleroglossans, suggesting that this common pattern may represent a primitive condition within Squamata [50]. On the other hand, the total scleral ossicle count in the derived mosasaurine *Mosasaurus* proved distinct from the two russellosaurine genera, and no known extant lizard taxa shared the ossicle arrangement of *Mosasaurus*, either. We here propose, based on all the osteological data known to us of mosasaurs, that russellosaurine *Tylosaurus* and *Platecarpus* likely exhibit evolutionary conservatism of the sclerotic ring structure among squamates, while mosasaurine *Mosasaurus* evolved a novel configuration. Given the mosaic distribution of the sclerotic ossicle counts and arrangement patterns among Squamata broadly and obligatorily aquatic mosasaurs specifically, these attributes are unlikely to have had any major effects on secondary aquatic adaptation in mosasaurs.

Finally, unlike many modern reptiles and birds, all examined mosasaur sclerotic rings consistently lack a sigmoid flexure along the internal margin of the ring. In addition, the inner side of the ring in *Platecarpus tympaniticus* exhibited a concentric ridge with distinctly asperate surface that, to our knowledge, no modern lizards are known to possess. These features possibly relate to adaptation for the underwater vision in mosasaurs, but this constitutes a hypothesis that requires further investigations using a number of modern taxa.

## References

[1] Jacobs LL, Ferguson K, Polcyn MJ, Rennison C (2005) Cretaceous δ13C stratigraphy and the age of dolichosaurs and early mosasaurs. Netherlands J Geosci 84: 257–268.

[2] Caldwell MW (1996) Ontogeny and phylogeny of the mesopodial skeleton in mosasauroid reptiles. Zool J Linn Soc 116: 407–436.

[3] Caldwell MW (2002) From fins to limbs to fins: limb evolution in fossil marine reptiles. Am J Med Genet 112: 236–249. 12357467 10.1002/ajmg.10773

[4] Carroll RL (1997) Mesozoic marine reptiles as models of long-term, large-scale evolutionary phenomena. In: Callaway JM, Nicholls EL, editors. Ancient Marine Reptiles. San Diego: Academic Press pp. 467–489.

[5] Lindgren J, Caldwell MW, Konishi T, Chiappe LM (2010) Convergent Evolution in Aquatic Tetrapods: Insights from an Exceptional Fossil Mosasaur. PLoSONE 5(8): e11998 10.1371/journal.pone.0011998 20711249 PMC2918493

[6] Lindgren J, Kaddumi HF, Polcyn MJ (2013) Soft tissue preservation in a fossil marine lizard with a bilobed tail fin. Nat Commun 4, Article number: 2423. 10.1038/ncomms3763 24022259

[7] Russell DA (1967) Systematics and morphology of American mosasaurs. Bull Peabody Mus Nat Hist 23: 1–241.

[8] Bell GL Jr, Polcyn MJ (2005) *Dallasaurus* turneri, a new primitive mosasauroid from the Middle Turonian of Texas and comments on the phylogeny of Mosasauridae (Squamata). Netherlands J Geosci 84: 177–194.

[9] Everhart MJ (2005) *Tylosaurus kansasensis*, a new species of tylosaurine (Squamata, Mosasauridae) from the Niobrara Chalk of western Kansas, USA. Netherlands J Geosci 84: 231–240.

[10] Marsh OC (1880) New characters of mosasauroid reptiles. Am J Sci 109: 83–87.

[11] Dollo L. (1889) Première note sur les mosasauriens de Mesvin. BullBelg. Geol 3: 271–304.

[12] Edinger T. (1929) Uber knocherne Scleralringe. Zool Jahrb Abt Anat 51: 163–226.

[13] Plisnier-Ladame F, Coupatez P (1969) Tude morphologique de l’anneau sclrotique de *Mosasaurus hoffmanni* Mantell, 1829. Bulletin Belgische Vereniging Geologie Paleontologie Hydrologie. 78: 253–265.

[14] Underwood G (1970) The eye. In: Gans C, Parsons TS, editors. Biology of the Reptilia, Volume 2, Morphology B. New York: Academic Press pp. 1–97.

[15] Goldfuss A (1845) Der schädelbau des *Mosasaurus*, durch beschreibung einer neuen art dieser gattung erläutert. Nova Acta Academa Ceasar Leopoldino-Carolinae Germanicae Natura Curiosorum 21: 1–28.

[16] Marsh OC (1872) Discovery of the dermal scutes of mosasaurid reptiles. Am J Sci 16: 290–292.

[17] Williston S. W. (1914). Water reptiles of the past and present. Chicago, Ill University of Chicago Press 251 p.

[18] Konishi T, Brinkman D, Massare JA, Caldwell MW (2011) New exceptional specimens of *Prognathodon overtoni* (Squamata, Mosasauridae) from the upper Campanian of Alberta, Canada, and the systematics and ecology of the genus. J Vertebr Paleontol 31: 1026–1046.

[19] Thewissen JG, Nummela S (2008) Sensory evolution on the threshold: adaptations in secondarily aquatic vertebrates. Univ of California Press 351 p.

[20] Vikaryous MV (2006) Skeletal elements in the vertebrate eye and adnexa—morphological and developmental perspectives. Review for a Special Issue on Craniofacial Development Developmental Dynamics 235: 1244–1255. 10.1002/dvdy.20718 16496288

[21] Lima FC, Vieira LG, Santos ALQ, De Simone SBS, Hirano LQL, et al (2009) Anatomy of the scleral ossicles in brazilian birds. Braz J Morphol Sci 26: 165–169.

[22] Lemmrich W (1931) Der Skleralring der Vögel. Jenaische Zeitschrift für Naturwissenschaft 65: 513–586.

[23] Romer AS (1956) Osteology of the Reptiles. Chicago, University of Chicago Press pp. 772

[24] Ott M (2006) Visual accommodation in vertebrates: mechanisms, physiological response and stimuli. J Comp Physiol A 192: 97–111. 16172892 10.1007/s00359-005-0049-6

[25] Coulombre AJ, Coulombre JL (1973) The skeleton of the eye: II. Overlap of the scleral ossicles of the domestic fowl. Dev Biol 33: 257–267. 4789605 10.1016/0012-1606(73)90136-x

[26] Schmitz L (2009) Quantitative estimates of visual performance features in fossil birds. J Morphol 270: 759–773. 10.1002/jmor.10720 19123246

[27] Hall M (2009) The relationship between the lizard eye and associated bony features: a cautionary note for interpreting fossil activity patterns. Anat Rec 292: 798–812. 10.1002/ar.20889 19462447

[28] Martin GR (1994) Form and function in the optical structure of bird eyes. In: Davies MNO and Green PR, editors. Perception and motor control in birds: an ecological approach. New York Springer pp. 5–34.

[29] Sivak JG (2004) Through the Lens Clearly: Phylogeny and Development The Proctor Lecture. Invest Ophthalmol Vis Sci 45: 740–747. 14985284 10.1167/iovs.03-0466

[30] Mass AM, Supin AY (2007) Adaptive features of aquatic mammals' eye. Anat Rec 290: 701–715. 17516421 10.1002/ar.20529

[31] Longrich N (2010) The function of large eyes in *Protoceratops*: a nocturnal ceratopsian. In: Ryan MJ, Chinnery-Allgeier BJ, Eberth DA, editors. New perspectives on horned dinosaurs: The Royal Tyrrell Museum Ceratopsian Symposium. Bloomington: Indiana University Press pp. 308–327.

[32] Schmitz L, Motani R (2011) Nocturnality in dinosaurs inferred from scleral ring and orbit morphology. Science 332: 705–708. 10.1126/science.1200043 21493820

[33] Motani R, Rothschild BM, Wahl W Jr (1999) Large eyeballs in diving ichthyosaurs. Nature 402: 747–747. 10.1016/s0002-9394(00)00518-3 10927017

[34] Humphries S, Ruxton GD (2002) Why did some ichthyosaurs have such large eyes? J Exp Biol 205: 439–441. 11893757 10.1242/jeb.205.4.439

[35] Konishi T, Caldwell MW, Bell GL Jr (2010) Redescription of the holotype of *Platecarpus tympaniticus* Cope, 1869 (Mosasauridae: Plioplatecarpinae), and its implications for the alpha taxonomy of the genus. J Vertebr Paleontol 30: 1410–1421.

[36] Nicholls EL (1988) Marine vertebrates of the Pembina Member of the Pierre Shale (Campanian, Upper Cretaceous) of Manitoba and their significance to the biogeography of the Western Interior Seaway. Unpublished Ph.D. dissertation, University of Calgary, Calgary, Alberta. 317 p.

[37] Konishi T, Caldwell MW (2011) Two new plioplatecarpine (Squamata, Mosasauridae) genera from the Upper Cretaceous of North America, and a global phylogenetic analysis of plioplatecarpines. J Vertebr Paleontol 31: 754–783.

[38] Konishi T, Newbrey MG, Caldwell MW (2014) A small, exquisitely preserved specimen of *Mosasaurus missouriensis* (Squamata: Mosasauridae) from the upper Campanian of the Bearpaw Formation, western Canada, and the first stomach contents for the genus. J. Vertebr Paleontol 34: 802–819.

[39] McNeil DH (1984) The eastern facies of the Cretaceous system in the Canadian Western Interior; In Stott DF, Glass DJ, editors. The Mesozoic of Middle North America. Canadian Society of Petroleum Geologists Memoir 9 pp. 145–171.

[40] Konishi T, Caldwell MW (2009) New material of the mosasaur *Plioplatecarpus nichollsae* Cuthbertson et al., 2007, clarifies problematic features of the holotype specimen. J Vertebr Paleontol 29: 417–436.

[41] Everhart MJ (2001) Revisions to the biostratigraphy of the Mosasauridae (Squamata) in the Smoky Hill Chalk Member of the Niobrara Chalk (Late Cretaceous) of Kansas. Trans Kans Acad Sci 104: 59–78.

[42] Williston SW (1898) Mosasaurs. Kans Univ Geol Survey, Paleontology 4/5:81–221.

[43] Hattin DE (1996) Fossilized regurgitate from Smoky Hill Member of Niobrara Chalk (Upper Cretaceous) of Kansas, USA. Cretaceous Research 17: 443–450.

[44] Obradovich JD (1993) A Cretaceous time scale. Evolution of the Western Interior Basin 39: 379–396.

[45] Konishi T, Lindgren J, Caldwell MW, Chiappe L (2012) *Platecarpus tympaniticus* (Squamata, Mosasauridae): osteology of an exceptionally preserved specimen and its insights into the acquisition of a streamlined body shape in mosasaurs. J Vertebr Paleontol 32: 1313–1327.

[46] Tsujita CJ (1995) Origin of concretion-hosted shell clusters in the Late Cretaceous Bearpaw Formation, southern Alberta, Canada. Palaios 10: 408–423.

[47] Tsujita CJ, Westermann GEG (1998) Ammonoid habitats and habits in the Western Interior Seaway: a case study from the Upper Cretaceous Bearpaw Formation of southern Alberta, Canada. Palaeogeogr Palaeocl 144: 135–160.

[48] Kauffman EG (1977) Geological and biological overview: Western Interior Cretaceous basin. The Mountain Geologist 14: 75–99.

[49] Hattin DE (1982) Stratigraphy and depositional environment of Smoky Hill Chalk Member, Niobrara Chalk (Upper Cretaceous) of the type area, western Kansas. Kansas Geol Survey, Bulletin 225. 108 p.

[50] Gauthier JA, Kearney M, Maisano JA, Rieppel O, Behlke ADB (2012) Assembling the squamate tree of life: perspectives from the phenotype and the fossil record. Bull Peabody Mus Nat Hist 53: 3–308.

[51] Torres-Carvajal O (2003) Cranial osteology of the Andean lizard Stenocercus guentheri (Squamata: Tropiduridae) and its postembryonic development. J Morphol 255: 94–113. 12420324 10.1002/jmor.10051

[52] Pianka ER, Vitt LJ (2003) Lizards: windows to the evolution of diversity. Univ of California Press 333 p.

[53] Walls GL (1942) The vertebrate eye and its adaptive radiation. Oxford, England: Cranbrook Institute of Science 785 p.

[54] Sivak JG (1975) Accommodative mechanisms in aquatic vertebrates. In: Ali MA, editor. Vision in fishes. Vision in Fishes. New York: Plenum pp. 289–297.

[55] Sivak J, Howland HC, McGill-Harelstad P (1987) Vision of the Humboldt penguin (*Spheniscus humboldti*) in air and water. P Roy Soc Lond B Bio 229: 467–472. 2881308 10.1098/rspb.1987.0005

[56] Brudenall DK, Schwab IR, Fritsches KA (2008) Ocular morphology of the Leatherback sea turtle (Dermochelys coriacea). Vet Ophthalmol 11: 99–110. 10.1111/j.1463-5224.2008.00607.x 18302574

[57] Land MF, Nilsson D-E (2002) Animal Eyes. New York, Oxford University Press 221 p.

